# siRNA Delivery Strategies: A Comprehensive Review of Recent Developments

**DOI:** 10.3390/nano7040077

**Published:** 2017-04-05

**Authors:** Katyayani Tatiparti, Samaresh Sau, Sushil Kumar Kashaw, Arun K. Iyer

**Affiliations:** 1 Use-Inspired Biomaterials & Integrated Nano Delivery (U-BiND) Systems Laboratory, Department of Pharmaceutical Sciences, Eugene Applebaum College of Pharmacy and Health Sciences, Wayne State University, Detroit, MI 48201, USA; katyayani.tatiparti@wayne.edu (K.T.); samaresh.sau@wayne.edu (S.S.); sushilkashaw@gmail.com (S.K.K.); 2 Department of Pharmaceutical Sciences, Dr. Harisingh Gour University (A Central University), Sagar 470003, India; 3 Molecular Therapeutics Program, Barbara Ann Karmanos Cancer Institute, School of Medicine, Wayne State University, Detroit, MI 48201, USA

**Keywords:** siRNA, nanoparticles, delivery systems, cancer therapy, tumor, RNAi, gene delivery, targeted delivery

## Abstract

siRNA is a promising therapeutic solution to address gene overexpression or mutations as a post-transcriptional gene regulation process for several pathological conditions such as viral infections, cancer, genetic disorders, and autoimmune disorders like arthritis. This therapeutic method is currently being actively pursued in cancer therapy because siRNA has been found to suppress the oncogenes and address mutations in tumor suppressor genes and elucidate the key molecules in cellular pathways in cancer. It is also effective in personalized gene therapy for several diseases due to its specificity, adaptability, and broad targeting capability. However, naked siRNA is unstable in the bloodstream and cannot efficiently cross cell membranes besides being immunogenic. Therefore, careful design of the delivery systems is essential to fully utilize the potential of this therapeutic solution. This review presents a comprehensive update on the challenges of siRNA delivery and the current strategies used to develop nanoparticulate delivery systems.

## 1. Introduction

RNAi was first discovered by Mello and Fire in mammalian cells. Since then it has led to great enthusiasm in the field of post-transcriptional silencing of gene expression. The RNAi mechanism was first discovered by *Caenorhabditis elegans* when an exogenously introduced dsRNA caused a systemic gene suppression [1]. It gave the idea that there might be an active intermediate that facilitated gene silencing [2]. These intermediates included the dicer enzymes and the RNA-induced silencing complex (RISC) which is a complex of proteins and the siRNA molecules with a highly conserved Argonaute protein, Argonaute-2 (AGO2) as the core.

**Figure 1:**
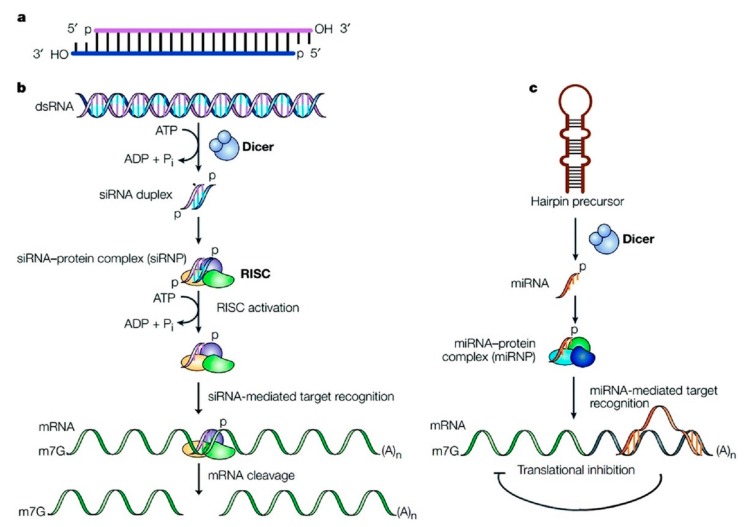
(**a**) Structure of siRNA; (**b**) siRNA pathway; (**c**) miRNA pathway (Reproduced with permission from [8]. Copyright the Royal Society of Chemistry, 2003).

The mechanism of siRNA mediated gene silencing can be said to have two main stages namely post-transcriptional gene silencing (PTGS) which can be classified further into two mechanisms called the direct sequence-specific cleavage leading to translation repression and consequent degradation, and transcriptional gene silencing (TGS) [3], both of which have a specific repression effect. In detail, the direct sequence specific cleavage mechanism can be described as follows: Endogenous dsRNA is identified by a ribonuclease protein called the dicer which cleaves it into small double stranded fragments of 21 to 23 base pairs in length with 2-nucleotide overhangs at the 3′ ends. These cleaved products have been recognized as the small interfering RNAs (siRNAs). They consist of a passenger strand and a guide strand, that are connected to each other by an active protein complex called the RNA-Induced Silencing Complex (RISC). After binding to RISC, the guide strand is directed to the target mRNA, to cleave it into small pieces that are between bases 10 and 11 relative to the 5′ end of the siRNA guide strand by the cleavage enzyme argonaute-2. Thus, the process of mRNA translation can be interrupted by siRNA [4,5,6,7]. The miRNA mediated pathway gene silencing mechanism involves affecting the mRNA stability by mediating its degradation and/or by inhibiting protein translation or interfering with the polypeptides through complimentary binding to 3′UTR of specifically targeted mRNAs. A summary of the processes is shown in Figure 1 [8].

**Figure 2:**
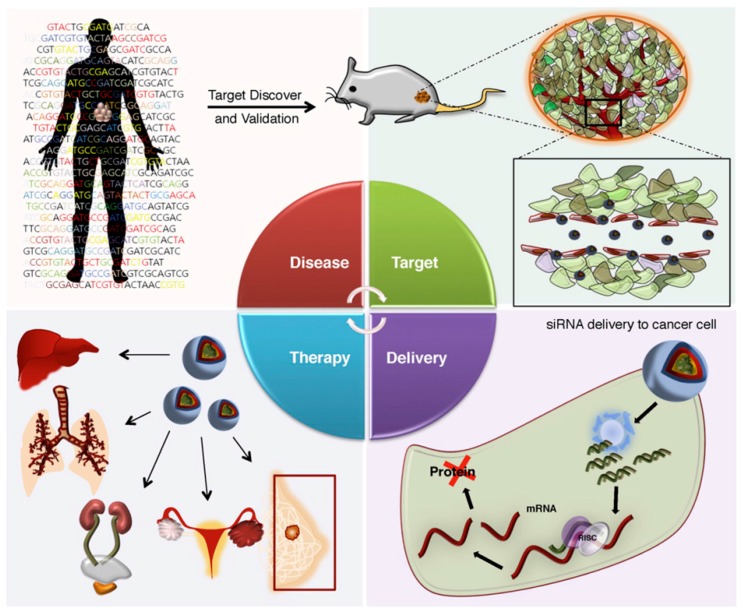
Designing siRNA therapeutics for cancer treatment (Reproduced with permission from [14], Copyright Elsevier, 2015).

This discovery was a rewarding milestone in gene therapy because it opened opportunities that could shine some light on several molecular pathways involved in several diseases like cancer, genetic disorders, autoimmune diseases, and viral infections. Cancer therapy, in particular, has already made use of this new-found knowledge in designing siRNA that can inactivate multiple gene mutations both in the oncogenes and the tumor suppressor genes that are the cause of cancer [9,10]. Following this strategical revelation, several synthetic siRNA are being designed with desirable sequences to apparently inhibit any target gene expression [11,12,13] (Figure 2 [14]). 

Making use of these unique features of siRNA, several delivery systems for siRNA have entered the clinical trial phase very recently and are being pursued as a very efficient and promising cure for cancer (Table 1 [15]). These delivery systems mostly aim at making the siRNA more efficient at interference with angiogenesis, metastasis, chemo-resistance of tumors, and the proliferation of cancer cells [16]. Though these systems are showing encouraging results for prospective commercial success, many obstacles still remain to practically apply them as therapeutics in humans.

## 2. Challenges in the Delivery of siRNA and Strategies to Address Them

**Figure 3:**
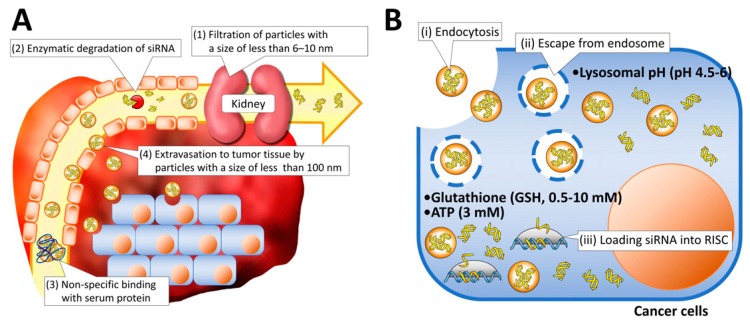
Schematic illustration of delivery barriers in extracellular (**A**) and intracellular (**B**) regions (Reproduced with permission from [18]. Copyright Elsevier, 2016.).

siRNA therapeutics apply the concept of ‘loss-of-function approach’ to treat cancer which involves limiting or preventing target protein expression within the cells, thus, altering the proliferation of cancer cells. Also, siRNA is not incorporated into DNA. Hence, the genome is not presented with the problem of permanent modification. This allows for the convenience to stop and control the siRNA therapy at any point in time and stage of treatment which fulfils a critical factor for regulatory and safety considerations [17]. However, the access to the full potential of siRNA therapy is limited by its ineffective delivery to the target systems. Figure 3 [18] summarizes the extracellular and the intracellular challenges seen while attempting the delivery of siRNA to targets.

There are several challenges presented to siRNA delivery such as efficient delivery of RNAi therapeutics to tumors after reaching the circulation by protecting them from enzymatic degradation and rapid renal filtration, entrapment by phagocytes, and extravasation from blood to tumor tissues. Upon reaching the tumor, it has to overcome the vascular barrier and be internalized by cancer cells by cellular uptake, and then escape from the endosome into the cytoplasm, and finally be released from the siRNA payload to form RISC [18]. The major challenges and the strategies to address them are described below.

### 2.1. Administrative Barrier

Most of the cancer target sites are not available by oral route. Also, this route is not good in maintaining intestinal stability and insufficient permeability across intestinal epithelium into circulation [19]. Another route of administration is the subcutaneous injection which has the advantage of bypassing the first-pass effect of the liver and access to the circulation via capillaries or lymphatic drainage from interstitial space. However, the lipophilicity and the size of the gene vectors are a challenge to be taken into consideration to avoid the phagocytosis of these molecules by the phagocytic cells of the immune system. The most common modes of administration are, thus, the intravenous or infusion injections.

### 2.2. Vascular Barrier

Crossing the vascular barrier is the crucial step for siRNA delivery in order for it to reach the targeted systems. Hence, the most beneficial feature in vasculature for successful delivery of siRNA is discontinuous sinusoidal capillaries with large openings that allow the leaking of siRNA nanocarriers into the blood stream. These capillaries are widely found in the liver. However, the size of the nanocarriers is a limitation here and has to be up to 100 nm. A similar morphology of capillaries is found in tumor cells besides considerable variation of cell composition, basement membrane, and pericyte coverage and this allows the access and the accumulation of the nanocarriers in the tumor cells by a phenomenon called the enhanced permeability and retention (EPR) effect [20]. Thus, when targeting the tumor, four factors are important to make the most of the EPR effect (1) internal and external blood flow of the tumor; (2) tumor vascular permeability; (3) structural barriers enforced by extracellular matrix and tumor cells; and (4) intra-tumoral interstitial pressure [21]. Longer half-life of the siRNA therapeutics is also crucial to tap the EPR effect efficiently [15]. For targeting the non-hepatic tumor sites, care should be taken to design the therapeutics because these capillaries have much smaller pores (60–80 nm in diameter) and the endothelium is covered with continuous basal lamina, which can prevent the diffusion of large-scale nanoparticles. In such cases, the delivery is evidently affected by the tightness, shape of the pores, continuous basal lamina, and the extracellular matrix.

Further, one of the main mechanisms of removal of the siRNA therapeutics from the bloodstream is through urine by glomerular filtration in the kidneys. The pore size of the glomerular filtration barrier is about 8 nm. So, if the nanoparticles are designed to have a particle size of about 20 nm, then this barrier challenge can be addressed efficiently [22,23,24].

Once the siRNA therapeutics reach the blood stream, they have to be protected from the phagocytic cells of the mononuclear phagocyte system (MPS) [25]. In this case, the important factors to be taken care of are formulation size, surface electrostatic nature, lipophilicity, and stability of the formulation. A large size of the formulation is undesirable since large particles are more susceptible to phagocytosis. Excessive net charge also elicits a similar response because they tend to aggregate due to electrostatic forces. Hence, the minimum net charge should be maintained through modification with hydrophilic and neutral molecules like PEG to increase the stability of the formulation in the blood stream [26]. However, care should be taken not to over-stabilize it by attaching targeting moieties so as to prevent the uptake by cells altogether. Further, it has been observed that increased lipophilicity allows more accumulation of the therapeutics in the tumor cells [27]. Hence, the therapeutics should be designed accordingly. Apart from this, the fact that tumor vascularity is controlled by the oxygen supply, some metabolites should be considered because then an increase in tumor vascularity can increase the efficiency of the siRNA delivery system [28,29]. 

### 2.3. Cellular Barriers

The next challenge is the cellular uptake of the therapeutics. The cellular membrane is made of negatively charged phospholipids in a bilayer consisting of functional proteins. This charge is a barrier for siRNA nanocarrier uptake. To overcome this challenge, mostly the means of endocytosis has been adopted [30] and in particular, targeted endocytosis such as receptor mediated endocytosis using ligands as folate [31], transferrin [32], and aptamers [33].

The next challenge would be endosomal escape for the successful approach of the therapeutic towards the RNA-Induced Silencing Complex (RISC) in cytoplasm. Ideally, the endosomal escape should happen before the late endosomes fuse with the lysosomes which contain digestive enzymes, a process which involves a gradual drop in the pH inside the endosome from the early endosomal stage to the lysosomal fusion stage [34,35]. There are two methods to achieve this. One is the use of cationic polymers to increase the endosomolysis by their acidification upon absorption of the protons and destabilization of their membranes. The other method is the rupture of the lysosome by increasing the uptake of protons, termed the proton sponge effect, such that the osmotic pressure inside the lysosome increases eventually rupturing it and releasing the therapeutics [36]. Yet another method is using neutrally charged ionizable lipids that become positively charged inside an endosome that leads to their disruption to release the siRNA carriers [37]. Nevertheless, the endosomal release system is poorly understood. So, more insight into this will open up a whole new opportunity for efficient siRNA delivery.

### 2.4. Immune Response and Safety

This is one of the important challenges that siRNA delivery systems have to address. The delivery systems are required to be non-immunogenic and they should not elicit undesirable side-effects. They should refrain from off-target silencing of the genes in normal cells [38,39]. They should not be identified as foreign particles by the innate immune system, especially the interferons and cytokines, in order to prevent being destroyed before reaching the target [40,41]. This challenge can be overcome by designing the siRNA which is between 21–23 base pairs in length [40]. The other method is to chemically modify siRNA by 2′-*O*-methylation to prevent an immune response in the body after administration. 

## 3. Role of Nanoparticles in siRNA Delivery

In order to address the above challenges, nanoparticles are the most common choice made in order to deliver the unstable naked siRNA to the targeted tumor sites since they protect the siRNA from plasmatic nucleases and undesirable immune responses thus assisting in endocytosis. Further, they can be used for targeted delivery by attaching target-specific ligands onto their surface. However, the utility of the nanoparticles is limited by their physicochemical properties which are summarized in Table 2 [1].

There are several advantages to the use of nanoparticles: (1) particle size is desirable for the purpose of siRNA delivery to overcome the barriers; (2) they are inert and hence non-immunogenic; (3) some can stimulate interferon-γ production and augment natural killer (NK) cells resulting in activation of antitumor immunity enhancing the efficiency of the therapy altogether; (4) they have enhanced circulation time allowing them to penetrate and accumulate in tumor cells more efficiently; (5) they can be imaged and tracked. On the other hand, they have certain disadvantages like (1) poor water solubility; (2) poor hydrophobicity; (3) they have limited bioaccumulation. However, these limitations can be overcome by selection of suitable polymers. Thus, the advantages outweigh the disadvantages.

## 4. Types of Nanoparticulate Delivery Systems for siRNA Therapeutics

### 4.1. Classification Based on the Material of Construct Used

**Figure 4:**
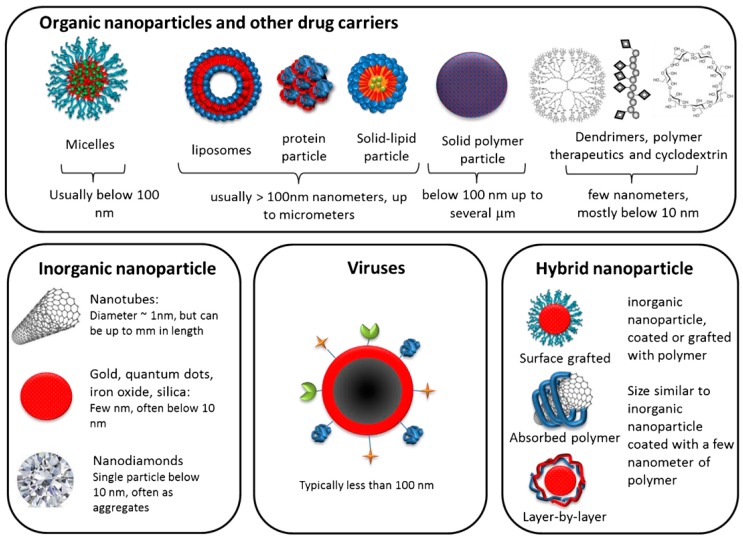
Nanoparticle illustrations that exist in both soft and hard (Reproduced with permission from [1]. Copyright Elsevier, 2016).

There are broadly two types of nanoparticles that are used in siRNA delivery for discussion; the soft/organic nanoparticles and the hard/inorganic nanoparticles. Often, the hard/inorganic nanoparticles are coated with polymers to manipulate their solubility. Sometimes, these may contain multiple coatings as per the requirement of the targeted delivery and hence can become complex. Another type of nanoparticles is the theranostic nanoparticles which will be discussed here. A simplified summary of these types of nanoparticles is shown in Figure 4 [1].

#### 4.1.1. Soft/Organic Nanoparticles

These are based on the usage of the organic material either from a natural or a synthetic source such as polymers or surfactants that are self-aggregating. The typical soft/organic nanoparticles include the liposomes, nanoemulsions, and dendrimers and polymer nanoparticles. Liposomes are the bilayer organic lipid molecules with different charges. While neutral liposomes are preferred, they have the limitation of poor entrapment efficiency [42]. Hence, zwitterionic 1,2-dioleoyl-sn-glycero-3-phosphatidylcholine (DOPC) is used.

#### 4.1.2. Hard/Inorganic Nanoparticles

These are the inorganic and insoluble nanoparticles that are non-biodegradable and biopersistent. Typical hard/inorganic nanoparticles are metals, metal oxides, and carbon materials (e.g., fullerenes, nanotubes, fibers) [43] and magnetic nanoparticles consisting of Super-Paramagnetic Iron Oxide Nanoparticles (SPIONs) [44]. Gold nanoparticles have the versatility of being used as a delivery system or therapeutic molecules themselves since they are anti-angiogenic and have antitumor properties that act by interfering with cellular processes. Quantum dots are the novel colloidal semiconductor nanocrystals which possess superior optical and electronic characteristics that can have antitumor effects by effectively delivering siRNA. Nanodiamonds are the most novel delivery systems being studied for siRNA therapeutics delivery [45]. Carbon nanotubes, in particular those with nanoneedles are being actively studied as delivery systems because of their cell-death-inducing activity [46].

#### 4.1.3. Theranostic Nanoparticles

These are the nanoparticles that include two or more nanostructures to form a hybrid nanoparticle that performs a theranostic function, i.e., both diagnostic and therapeutic functions [47,48]. This may include the use of both organic and inorganic materials for both storage and diagnostic and therapeutic functions. These materials help in reducing undesirable side effects and also monitor the therapy in vivo.

### 4.2. Classification Based on Function

#### 4.2.1. Carrier Design for Stability and Release

**Figure 5:**
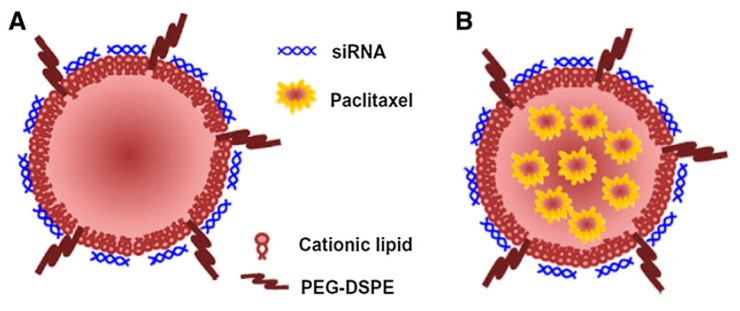
Multimolecular structures have a hydrophobized core (Taken with permission from [52]. Copyright Elsevier, 2014).

**Figure 6:**
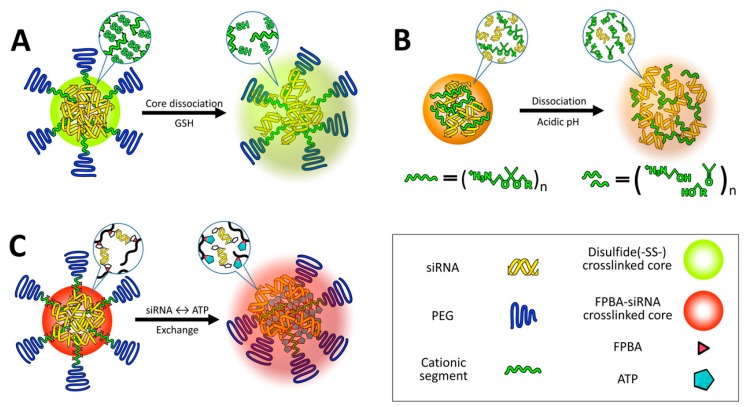
Multimolecular structures introduce redox-responsive thiol bonds in the core region (**A**); Delivery vehicle contains acidic pH responsiveness for faster dissociation (**B**) and introduce ATP-responsiveness in the core region for selective dissociation (**C**) (Reproduced with permission from [18]. Copyright Elsevier, 2016).

These delivery systems aim at increasing the delivery efficiency of the system by means of multimolecular delivery vehicles that are based on chemical structures and length of building components (cationic moieties, non-ionic/hydrophilic moieties, and hydrocarbon tails of lipids) [37,49]. An example of such a system is the ionizable cationic lipid in SNALP containing a dimethylamino headgroup (pKa = 6.7 ± 0.1) used to formulate siRNA as a multimolecular assembly at pH 4 where it maintains a neutral or low cationic surface charge density to avoid non-specific disruption of plasma membranes. This system can target specific sites based on three signals—three biological signals: redox potential, pH, and ATP concentration. The strategy for designing these systems is covalent conjugation of siRNA into delivery vehicles through biosignal-responsive crosslinkers; and the construction of multimolecular assembly using biosignal responsive components. Both these systems dissociate into building components and release siRNA. These can be designed as (1) hydrophobicity-stabilized delivery vehicles (made of hydrophobic moieties, such as alkyl chains and cholesterol, installed into cationic components) [18,50,51] as depicted in Figure 5 [52]; (2) delivery carrier design for selective release of siRNA such as the redox potential responsive delivery vehicles that involves glutathione conjugate systems [18,53,54,55], acidic pH responsive delivery vehicles which are based on the large difference in the extracellular (neutral) and endosomal pH (acidic) that uses acid-labile chemistry like the acetal, hydrazone, orthoester chemistry, etc., or by using phenylboronic acid (PBA) chemistry [18], and ATP concentration-responsive delivery vehicles that use the ATP signals to respond and release the siRNA as depicted in Figure 6 [18].

#### 4.2.2. Delivery Carrier Design for High Cell Specific Recognition

**Figure 7:**
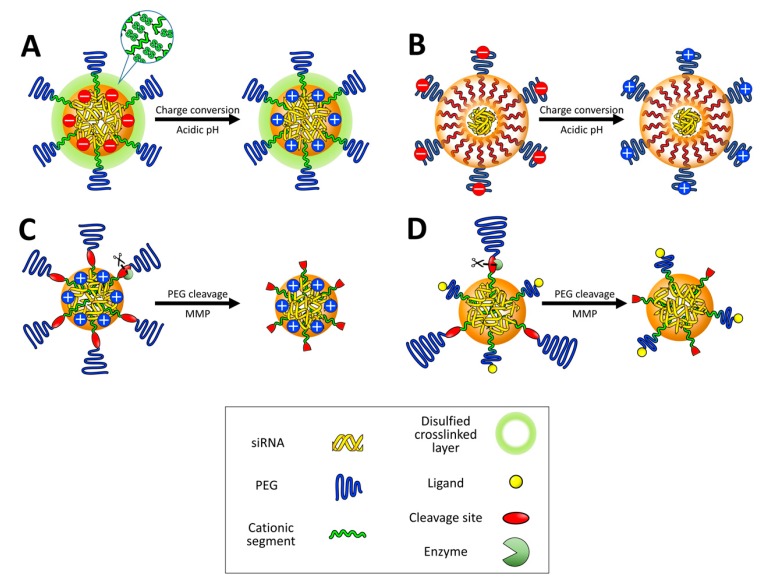
Multimolecular structures contain charge-conversion moieties in the core (**A**) or on to the surface (**B**); Enzymes e.g., (matrix metalloproteinases) cleave PEG layers, which exposes positive charges in the core (**C**) or cell-specific ligands (**D**) (Reproduced with permission from [18]. Copyright Elsevier, 2016).

These delivery systems involve a targeted and specific delivery mechanism that transports the siRNA therapeutics from the blood to the tumor site via blood vessels. In order to achieve this, these systems are required to be very stable in the blood. This, in turn, is accomplished by ligand–receptor interaction (or active targeting) with the cell-specific ligands attached on the surface of delivery vehicles or the distal ends of neutral and hydrophilic spacers. Another method that is practiced in this case involves subjecting the delivery vehicle to a selective exposure to the positive charges near target cells, facilitating the binding to the cellular surface. This means that the release of siRNA from these systems is in response (in terms of changing the multimolecular structures to release siRNA) to the tumor environment-specific biosignals such as acidic pH and specific enzymes as depicted in Figure 7 [18]. These systems can depend on various kinds of biosignals: (1) biological stimuli-responsive delivery vehicles that act at acidic pH, low oxygen levels, high carbon dioxide or more protons [18,56,57,58] and removal of the shielding layer due to any of these reasons [18,59,60,61,62,63]; (2) ligand installed delivery vehicles which involve attachment of ligands to the surface of the carrier and the specificity depends on the density, length/density of spacer like the GalNAc (tri-*N*-acetylgalactosamine) [18,64] and PEG [18,65], charge on the ligand and the size of the vehicle. 

#### 4.2.3. Delivery Vehicles for High Endosomal Escapability

The purpose of these systems is to produce efficient endosomal esacape and endosomal acidification. This is accomplished by the addition of groups like secondary/tertiary amines or histidine, PEI (polyethylenimine) (even though it is cytotoxic), etc., on the delivery vehicles [14,58,59,60]. These systems act by a proton sponge mechanism [18,36].

#### 4.2.4. Delivery Carrier Design in Other Categories

**Figure 8:**
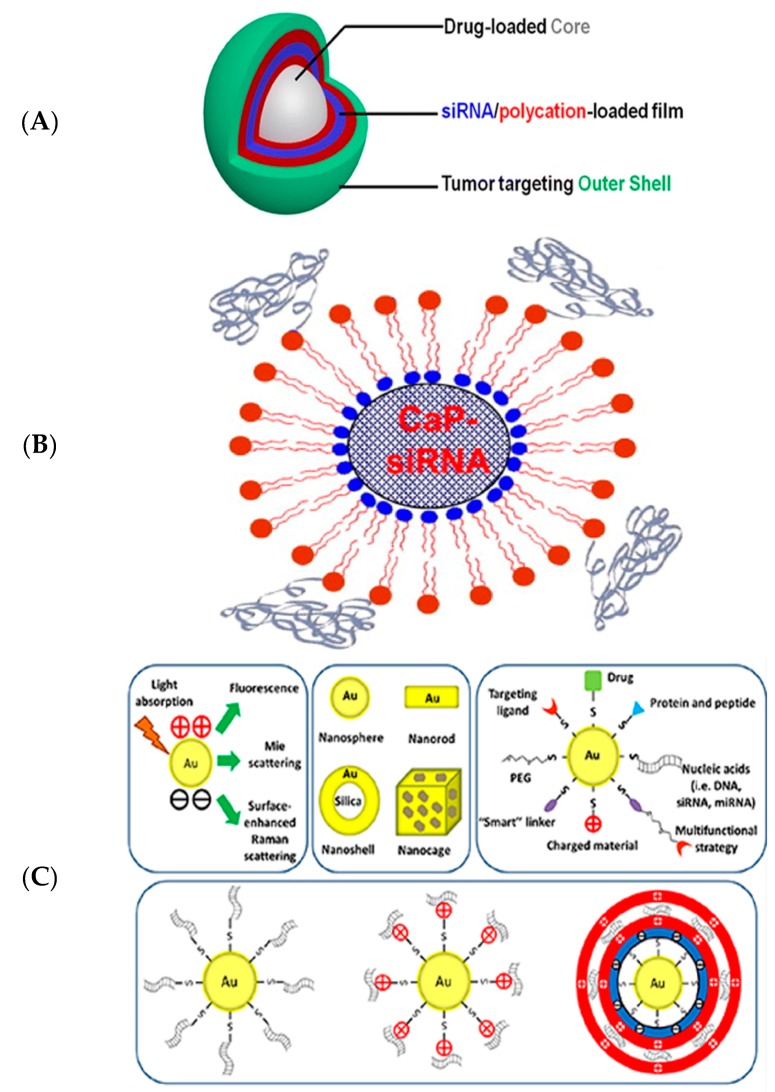
An LbL particle is constructed by alternative deposition of anionic and cationic components (**A**); A calcium phosphate particle stably formulates siRNA and PEG-polyanions in the core region (**B**); Thiolated siRNA is attached to Au nanoparticles and released in response of GSH (Glutathione) concentration (**C**) (Reproduced with permissions from [79,80,81]. Copyright ACS Nano, Elsevier and Springer, 2012, 2015 and 2013, respectively).

These systems involve miscellaneous strategies [66,67,68] like (1) the layer-by-layer delivery vehicle that is applied mostly for local delivery (though they are limited by their size) [18,69,70]; (2) calcium phosphate-formulated delivery vehicles that involve the co-deposition of inorganic molecules on the delivery systems for better size control and higher siRNA loading [18,71,72,73,74,75]; (3) Gold nanoparticle-templated delivery vehicles that use thiolated gold particles on the delivery systems to release siRNA [18,76,77], and mesoporous nanoparticles for siRNA delivery [78]. These systems have been comprehensively depicted in Figure 8 [79,80,81].

## 5. Conclusions

Cancer is still the second leading cause of deaths all over the world. There have been many advances in the gene sequencing of cancer cells that have led to the development of synthetic siRNA for delivering personalized medicine. Since their discovery, siRNAs therapeutics have been pursued actively because of their high specificity, easy modification, and unlimited therapeutic targets. However, being unstable in the blood, this presents several challenges with designing delivery systems for administration to the target sites. These challenges being both intracellular and extracellular are unavoidable. Because of these challenges, the utilization of this versatile therapeutic molecule to its full potential is still a long way away. Development of the various nanoparticulate systems described in this review, besides the other delivery systems that are being developed beyond the nanoparticulate systems, is an illustration of the extensive research progress taking place to accomplish targeted delivery of siRNA. Nanoparticulate systems have proven their worth and are widely recognized as the best means to achieve safe and targeted delivery of siRNA. However, there is much progress still needed for curing cancer using siRNA, especially in addressing the challenges discussed in this review. Continued research to understand the barriers that are still not fully known is one of the first steps that must be undertaken.

## Figures and Tables

**Table 1:** Current clinical status of RNAi therapeutics for cancer treatment (Taken with permission from [15]. Copyright Elsevier, 2015).

Indications	Name	Delivery Route	Target	Delivery System	Development Phase	Reference
Advanced solid tumors	siRNA-EphA2-DOPC	Intravenous (I.V) injection	EphA2	Lipid-based nanoparticles	Preclinical	NCT01591356
Metastatic tumors or cannot be removed by surgery	APN401	I.V injection	E3 ubiquitin ligase Cbl-b	Ex vivo transfection	Preclinical	NCT02166255
Metastatic melanoma, absence of CNS Metastases	iPsiRNA	Intradermal injection	LMP2, LMP7, MECL1	Ex vivo transfection	Phase I, completed	NCT00672542
Advanced solid tumors	Atu027	I.V infusion	PKN3	Lipid-based nanoparticles	Phase I, completed	NCT00938574
Pancreatic ductal adenocarcinoma; Pancreatic cancer	siG12D LODER	Intratumoral implantation	KRASG12D	LODER polymer	Phase I, completed	NCT01188785
Primary or secondary liver cancer	TKM-080,301	Hepatic intra-arterial injection	PLK1	Lipid-based nanoparticles	Phase I, completed	NCT01437007
METAVIR F3–4	ND-L02-s0201	I.V injection	HSP47	Lipid-based nanoparticles	Phase I, recruiting	NCT02227459
Solid tumors; multiple myeloma; non-Hodgkin’s lymphoma	DCR-MYC	I.V infusion	MYC	Lipid-based nanoparticles	Phase I, recruiting	NCT02110563
Cancer; solid tumor	CALAA-01	I.V injection	RRM2	Cyclodextrin-containing polymer	Phase I, terminated	NCT00689065
Neuroendocrine tumors; adrenocortical carcinoma	TKM 080301	I.V infusion	PLK1	Lipid-based nanoparticles	Phase I/II, recruiting	NCT01262235
Solid tumors	ALN-VSP02	I.V injection	KSP, VEGF	Lipid-based nanoparticles	Phase I, completed	NCT01158079NCT00882180

**Table 2:** Physicochemical characteristics of nanoparticulate delivery systems (Taken with permission from [1]. Copyright Elsevier, 2016).

Characteristics of Nanoparticles	Benefits of Optimizing Characteristics
Shape of particles	Improved rate of endocytosis allowing for a therapeutic dosage within the target cell
Size of particles	Avoidance of clearance and filtration by renal and hepatic systems therefore improving the half-life of the drug. This will allow it to remain within the therapeutic dosage for a longer period
Surface properties of particles	A suitable surface characteristic will allow for improved cellular uptake and prevent recognition from the immune system that may result in elimination of the nanoparticle
Thermal stability	To improve the stability of the conjugated siRNA to the nanoparticle to help prevent premature degradation and release of siRNA
pH stability	Allows for controlled release of nanoparticles within target cells and improved structural stability of the nanoparticle
Quality control	To prevent toxic events and side effects as contamination is prevented additionally it ensures the nanoparticle delivery is of a therapeutic standard
siRNA loading efficiency	This will determine the amount of siRNA the nanoparticle is able to carry a higher efficiency would require less nanoparticles for delivery and hence reduced side effects
